# Correlation between Tissue Characterization and Dynamic Expression of Matrix Metalloproteinase-2 and Its Tissue Inhibitor in Conjunctival Filtering Bleb of Rats

**DOI:** 10.1155/2017/1054129

**Published:** 2017-09-18

**Authors:** Ling Wang, Da-Bo Wang, Meng-Ying Liu, Ru-Yong Yao

**Affiliations:** ^1^Department of Ophthalmology, Affiliated Hospital of Qingdao University, Qingdao, China; ^2^Department of Ophthalmology, Shandong Provincial Hospital Affiliated to Shandong University, Jinan, China; ^3^Central Laboratory, Affiliated Hospital of Qingdao University, Qingdao, China

## Abstract

**Purpose:**

Using rat conjunctival bleb model, we correlated changes morphology and histology in the bleb with changes in MMP-2 and TIMP-2 levels.

**Methods:**

Filtering surgeries were performed on rats. Dynamic changes in morphology and histopathology were observed using HE staining. Expression of MMP-2 and TIMP-2 was determined by immunofluorescence microscopy and western blotting.

**Results:**

Well-elevated filtering blebs formed and persisted for an average of 12 days. Histological examination showed that inflammatory was dominant in postoperative days 1–3, and proliferating manifestation became the main sign 5 days later. Western blot showed that MMP-2 was downregulated 1 day after surgery, upregulated at 3 days, and observed with a peak at 7 days; then it persisted until 28 days. The difference was statistically significant (*F* = 280.18, *p* < 0.01).TIMP-2 was upregulated 1 day after surgery and observed with a peak at 5 days; then it persisted until 28 days. The difference was statistically significant (*F* = 145.34, *p* < 0.01).

**Conclusions:**

During the processes of conjunctival filtering bleb and scar formation in rats, the changes in MMP-2 and TIMP-2 levels in the filtering area, together with a corresponding proliferation of fibroblasts and the accumulation of collagen fibres, resulted in scarring of filtering blebs.

## 1. Introduction

Glaucoma is the second leading cause of blindness worldwide [1]. Elevated intraocular pressure (IOP) is considered the most consistent risk factor for glaucoma [2]. Filtration surgery is currently one of the most effective methods to treat glaucoma; the objective of this surgery is the creation of a fistula at the limbus, which allows the aqueous humour to drain from the anterior chamber into the subconjunctival space, thereby circumventing any pathological obstruction to outflow. A successful trabeculectomy is characterized by an elevation of the conjunctiva at the surgical site, commonly referred to as a filtering bleb [3]. It is essential that the filtering bleb remains functional; however, this may be complicated by the wound healing process after filtration surgery. Due to overhealing of the subconjunctival space at the bleb and sclerotomy sites, the failure rate of this surgery can reach 15%–30% [4, 5]. The cellular events related to extracellular matrix (ECM) deposition during wound healing play prominent roles in the outcome of filtration surgery [6]. During wound healing, the formation and remodeling of the ECM involve a series of events that occur in a highly sequential fashion [7]. Matrix metalloproteinases (MMPs) and their naturally occurring inhibitors, known as tissue inhibitor of matrix metalloproteinases (TIMPs), were shown to participate in connective tissue remodeling [8, 9].

Many scholars have focused on the expression of MMPs and TIMPs after glaucoma filtering surgeries. McClusky et al. examined the expression of MMPs and TIMPs in the blebs forming around Molteno implants by immunocytochemistry [10]. They found that MMP-1, MMP-2, MMP-3, and TIMP-2 were expressed in the filtering areas. Animal models of filtering blebs similarly showed increased MMP expression at the wound site. Shima et al. established a rabbit model of filtering surgery to study the expression of MMPs, analysing both protein and mRNA levels [11]. These studies detected the expression of MMP-1, MMP-2, MMP-3, and MMP-9 in the filtering areas. Although previous studies have suggested that MMPs may play an important role in the postoperative healing process at the wound site [12–14], our knowledge of these processes remains incomplete. We wished to explore any potential correlation between the tissue characterization and the dynamic changes of MMPs and their inhibitors during bleb formation and postoperative scarring. Studies have found significant increases in MMP-2 and MMP-9 activities after filtration surgery, which may play an important role in healing [7, 8]. Daniels et al. measured the expression of MMP-1, MMP-2, and MMP-3 in the in vitro collagen system of cultured fibroblasts. The expression of MMP-2 was the highest, suggesting that it may play a dominant role in scarring after filtration surgery [11]. Therefore, we selected MMP-2 and its tissue inhibitor, TIMP-2, as the subjects of our study. In this study, we established a mature and stable conjunctival bleb model in rats. We used this model to observe the morphological and histological changes in bleb formation and scarring processes and detected the changes of MMP-2 and TIMP-2 expression during these processes.

## 2. Materials and Methods

### 2.1. Surgical Procedures

All protocols in this study were both reviewed and approved by the Animal Research Committee of Affiliated Hospital of Medical College Qingdao University. All surgeries were performed under 10% chloral hydrate anaesthesia, additional topical anaesthesia was provided in the form of 0.4% oxybuprocaine hydrochloride drops, and all efforts were made to minimize suffering. Sixty-three male Sprague-Dawley adult rats (6–8 weeks), each weighing 350–400 g, were used in this experiment. Filtering surgeries were performed on 54 Sprague-Dawley rats by placing a drainage tube into the anterior chamber. In each of these rats, one eye was randomly selected for the experimental procedure, whereas the other eye received no treatment. Nine additional rats were selected as controls. Surgical anaesthesia was induced using 10% chloral hydrate, initially via an induction chamber, and subsequently delivered through a standard rodent nonrebreathing anaesthetic circuit connected to the animal by facemask. Additional topical anaesthesia was provided in the form of 0.4% oxybuprocaine hydrochloride drops. Cannulated filtering surgeries were performed as described by Sherwood et al. [15]. Briefly, a limbus-based conjunctival flap was created 3-4 mm behind the limbus by making a conjunctival incision and elevating the underlying Tenon's capsule using blunt dissection. A full-thickness scleral tunnel was then created using a 29-gauge needle, which was inserted into the anterior chamber. A microtubule of silicone, with an inner diameter of 0.3 mm, outer diameter of 0.6 mm, and length of ~3-4 mm, was then inserted through the sclera tunnel. Because the embedded pipe and the sclera tunnel connection are relatively close, we did not need to suture a fixed position. The proximal end of the cannula was trimmed flat approximately 1 mm behind the limbus and a “bead” of fluid was visualized to confirm patency. The conjunctiva and Tenon's capsule were closed in a single layer, using a nonabsorbable 10-0 nylon suture material attached in a continuous locking pattern. In all cases, a filtering bleb was observed immediately after surgery. A single drop of erythromycin eye ointment was applied postoperatively as an anti-inflammatory.

#### 2.1.1. Postoperative

Following surgery, the presence of a bleb (elevation of the conjunctiva) was noted and the day that the bleb could no longer be distinguished was scored as the time of failure. The main outcome measures we observed were bleb morphology, changes in the anterior chamber, lens turbidity, and the general health of the animal. We also noted eyelid swelling, conjunctival hyperaemia healing, and corneal conditions. The postoperative bleb could be avascular or include bleb surface blood vessels, which were scored as normal blood vessels, vessels with congestion, or vessels with severe congestion. We recorded functional bleb survival time according to the Kronfeld bleb morphology and functional classification [16]. After that, diffused elevated blebs with or without microcysts or avascular or slight hyperemia were recorded as functional filtering blebs. Localized elevated blebs in operation area with encapsulation or corkscrew vessels or massive hyperemia were recorded as nonfunctional filtering blebs.

### 2.2. Materials and Samples

Rats were sacrificed for enucleation at 1, 3, 5, 7, 14, and 28 days following surgery, so 54 rats with filtering surgeries were randomly divided into 6 groups according to the above time points, 9 rats in each group. The method of sacrifice of rats was administered with pentobarbital sodium 200 mg/kg by intraperitoneal injection which can produce a breath stop; then the experimental animal heart beating was checked. We examined 9 eyes at each time point and observed the process of formation and scarring of filtering blebs. Nine additional rats without filtering operation were killed at the same time points and used as controls. Once the rats were sacrificed, the operated eyes were immediately enucleated. For each time point, 3 eyes were immediately snap-frozen, embedded in OCT compound (Miles Laboratories, Elkhart, IN), mounted on slides, and stored at −80°C. The eyes were then used for histopathological examination and detection of MMP-2 and TIMP-2 expression sites by immunofluorescent staining. The filtration conjunctival tissues of the 6 remaining eyes were used for protein level analysis, performed by western blot (see below). For these 6 eyes in each group for western blot, we measured intraocular pressure (IOP) with rebound tonometer (suowei-500, China) before rats were sacrificed. We could not observe IOP in 3 eyes in each group for histopathological examination and immunofluorescent staining because of inconvenience in our immunohistochemical lab.

### 2.3. Localization of MMP2 and TIMP2 Expression

After careful dissection, eyes were rinsed with PBS and embedded in OCT. Frozen sections (6 *μ*m) were cut at −20°C using a cryostat (Leica CM1950; Leica Biosystems, Germany), mounted on slides, and stored at −80°C until needed. Some slices were processed for HE staining with routine light microscopy for morphology and histopathology characterization. The left sections were fixed with 4% paraformaldehyde for 15 min at room temperature, washed twice with PBS containing 0.025% Tween-20 for 5 min, and blocked with 5% bovine serum albumin (BSA) and 0.5% Triton X-100 in PBS for 30 min at room temperature. Samples were incubated with primary antibodies [anti-MMP2 (ab37150, 1 : 200; Abcam) and anti-TIMP-2 (ab1828, 1 : 20; Abcam)] overnight at 4°C, followed by secondary antibody incubation with goat anti-rabbit IgG (CW0103; 1 : 100, cwbio) and goat anti-mouse IgG (CW0145, 1 : 100; cwbio), both Cy3 conjugated, for 1 h at 37°C. We performed 4,6-diamidino-2-phenylindole (DAPI) staining for 5 min to identify cell nuclei (1 : 4000; China Beyotime Institute of Biotechnology). Negative controls were performed for each immunofluorescence staining experiment by skipping the incubation with the primary antibody, and all lacked staining. The sections were visualized and photographed using a fluorescence microscope (DFC480, Leica).

### 2.4. Measuring Expression of MMP-2 and TIMP-2

The expression levels of MMP-2 and TIMP-2 were examined by western blot analysis. Tissues of interest were dissected with clean tools, snap-frozen, and kept on ice for immediate homogenization. In a tube, ~60 *μ*L of ice-cold lysis buffer per ~1 mg of tissue was added, and samples were homogenized using an electric homogenizer. The blade was rinsed twice with 200 *μ*L of lysis buffer, and the sample was kept at constant agitation for 2 h at 4°C. After centrifuging for 20 min at 12,000 rpm at 4°C, the tubes were placed on ice and the supernatant was aspirated to a fresh tube. Protein quantification of the supernatant was performed by the Bradford method. Equal amounts of total protein from each sample were separated by 10% SDS-PAGE and transferred to a PVDF membrane. After blocking with 10% nonfat dry milk in PBS containing 0.1% Tween-20, for 1 h at room temperature, membranes were probed with anti-MMP-2 (1 : 800; Santa Cruz) or anti-TIMP-2 (1 : 800; Santa Cruz) mouse monoclonal antibody, overnight at 4°C. This was followed by incubation with HRP-conjugated goat anti-mouse IgG (1 : 6000, Santa Cruz) secondary antibody for 1 h at room temperature. Blots were probed in parallel with an anti-GAPDH antibody as a loading control. Protein bands were visualized with an enhanced chemiluminescence kit (Enlight Western Blotting Reagents, Engreen Biosystem). Band intensities were quantified using Image-Pro Plus software (software serial number: 41M60032-00032, Media Cybernetics Company).

### 2.5. Statistical Analysis

All results are expressed as means ± standard error of the mean (SEM). Statistical analysis of western blot data was conducted using the SPSS software version 17.0 (software serial number: 5296295381, US SPSS Company), to determine the MMP-2 and TIMP-2 expression at each time point. We used the Shapiro-Wilk test for normal distribution of the data and Levene's test to verify the homogeneity of variance between the groups. The expression levels of MMP-2 and TIMP-2 were compared among groups using a one-way ANOVA. Results were considered significant when *p* < 0.05.

## 3. Results

### 3.1. General Conditions and Anterior Segment Observation following Surgery

Following filtering surgeries, animals were examined for the presence of a bleb and further monitored for time of failure. A shallow anterior chamber occurred in 4 eyes (incidence of 6.3%), and these animals were discontinued from the experiment. Filtering surgeries were performed in the remaining rats. Hyphemas were observed in 3 eyes (incidence of 4.7%); however, they were completely self-absorbed after 2-3 days. Varying degrees of aqueous flare and presence of cells in the aqueous humour disappeared 2–4 days after surgery. Bleb leak, bleeding, infection, and cataract endophthalmitis complications did not occur.

### 3.2. Intraocular Pressure

At day 1, IOP decreased significantly and presented slow upward trend at days 3 and 5, reached that before operation until day 7, and then persisted in this level at days 14 and 28. The IOP changes were statistically significant (*F* = 4.382, *p* < 0.01). Comparisons between IOP at days 1, 3, 5 and control group showed a statistically significant difference (*p* < 0.01, 0.05, and 0.05, resp.). The groups examined 7 days, 14 days, and 28 days after surgery were not significantly different from control group (*p* > 0.05, all). IOP changes at each time point are indicated in Table 1.

### 3.3. Bleb Survival Time

Filtering surgery and closure of the incised conjunctiva with a nylon suture produced an immediate conjunctival drainage bleb in all rats, an elevation that remained visible under slit lamp observation from postoperative days 7–17, with a mean of 12 days for failure. Avascular blebs were observed for the initial 24–48 h following surgery and became vascularized by postoperative days 4-5. The functional bleb survival rate was 77.7% at day 7; however, only 20% of the functional blebs were observed at day 14. The bleb survival at each time point is indicated in Table 2.

### 3.4. Bleb Morphology and Histopathology

**Figure 1:**
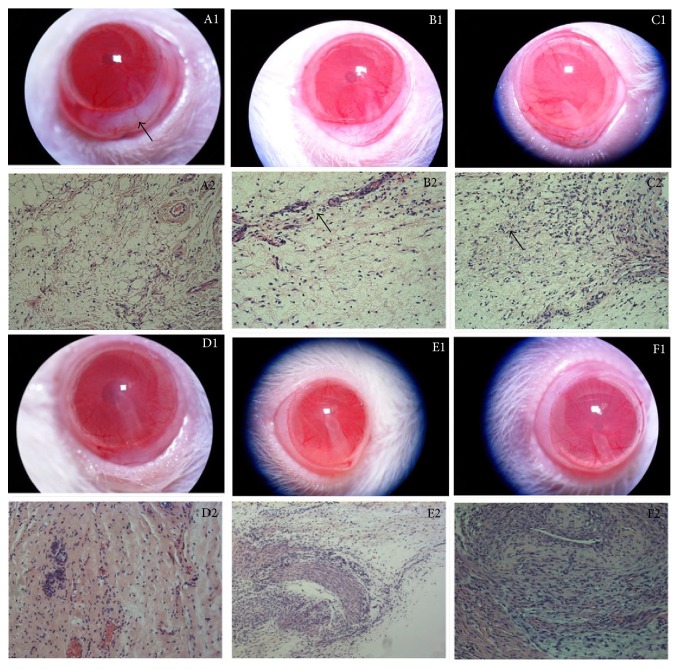
The morphology and histopathology in the conjunctival and subconjunctival tissue of the filtration area (HE staining A2, B2, C2, D2, F2: 200x amplification; E2: 100x amplification).

From days 1–3, the conjunctival blebs were elevated under slit lamp observation. HE staining revealed that the connective tissue of the bulbar conjunctiva was markedly oedematous and loose, with vascular dilation and obvious congestion and containing an abundance of infiltrating neutrophils. At day 5, conjunctival blebs were also elevated with vessels at the surface. Observation under the light microscope revealed that the oedematous connective tissue of the bulbar conjunctiva was unremarkable, that vascular dilation was alleviated, and that fibroblasts proliferated. At day 7, the conjunctival blebs appeared diffused. HE staining revealed vascular dilation and congestion with an abundance of fibroblasts and a few scattered, loosely meshed collagen fibres surrounding the conjunctival blebs. From days 14–28, conjunctival blebs became flat and gradually disappeared. HE staining revealed collagen proliferation in the area of surgery, forming the scar (Figure 1).

- At day 1: (1) blebs were elevated and with significant hyperemia (↖ indicates elevated bleb); (2) the connective tissue of the bulbar conjunctiva was markedly oedematous and loose and vascular dilation and congestion were obvious, with an abundance of infiltrating neutrophils.
- At day 3: (1) blebs were also elevated but hyperemia was alleviated; (2) the connective tissue of bulbar conjunctiva oedematous was loose and alleviated, vascular dilation and congestion were visible, and there was a scattered infiltration of neutrophils and macrophages (↖ indicates neutrophils).
- At day 5: (1) there were vessels at the blebs' surface; (2) the oedematous connective tissue of bulbar conjunctiva was unremarkable, there was infiltration of neutrophils and macrophages, and fibroblasts proliferated (↖ indicates fibroblasts).
- At day 7: (1) blebs were flat and elevated; (2) vascular dilation and congestion were obvious, with an abundance of fibroblasts.
- At day 14: (1) the blebs were only present in the surgery area; (2) the fibroblasts became reduced and morphologically slender. There was a visible increase in loose collagen.
- At day 28: (1) blebs almost disappeared; (2) the scar was composed mainly of mature and dense collagen, oriented in a parallel fashion with few fibroblasts.

### 3.5. Quantification of MMP-2 and TIMP-2 Expression

**Figure 2:**
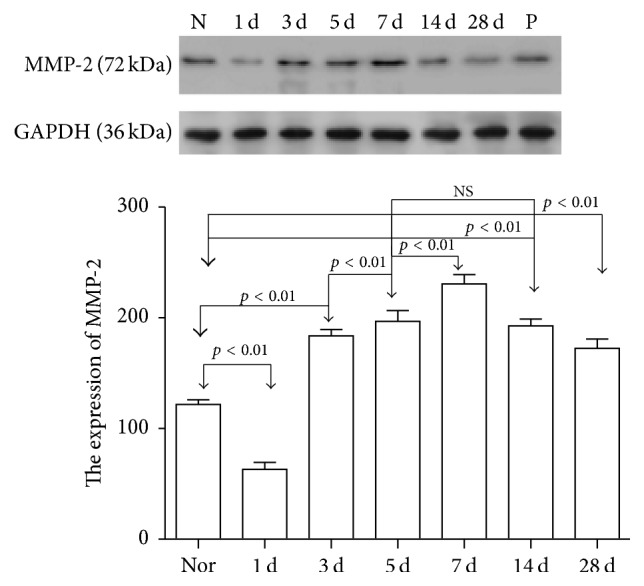
The expression of MMP-2 in conjunctival and subconjunctival tissue of filtration area. The values represent the mean ± SE of densitometry scans resulting from a western blot procedure. Numbers above bars represent levels of statistical significance among the groups. NS: not significant (N: control group; 1 d: at day 1; 3 d: at day 3; 5 d: at day 5; 7 d: at day 7; 14 d: at day 14; 28 d: at day 28; P: positive control).

**Figure 3:**
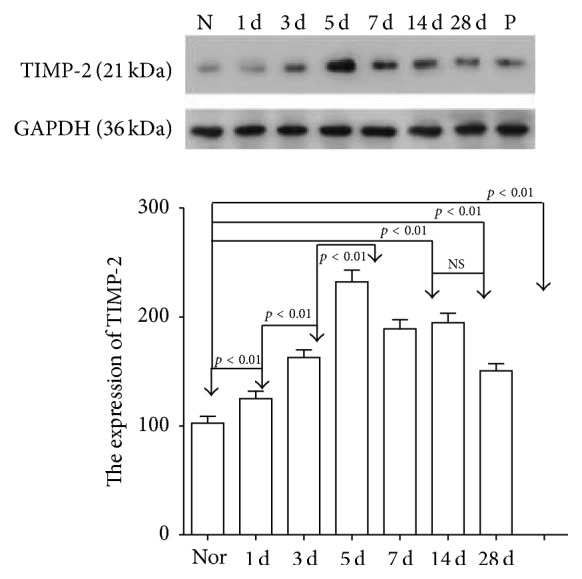
The expression of TIMP-2 in conjunctival and subconjunctival tissue of filtration area. The values represent the mean ± SE of densitometry scans resulting from a western blot procedure. Numbers above bars represent levels of statistical significance among the groups. NS: not significant. (N: control group; 1 d: at day 1; 3 d: at day 3; 5 d: at day 5; 7 d: at day 7; 14 d: at day 14; 28 d: at day 28; P: positive control).

We determined the expression levels of MMP-2 (Table 3, Figure 2) and TIMP-2 (Table 4, Figure 3) in conjunctival and subconjunctival tissue at different time points. The internal reference average grey value was 498.38 ± 19.53. At day 1, MMP-2 was reduced to about half of the normal level. Starting on day 3, MMP-2 expression increased, reaching its peak on day 7, and then gradually decreased until day 28 to values that were still higher than those measured for the control group. The increases in MMP-2 expression in conjunctival and subconjunctival tissues were statistically significant (*F* = 280.18, *p* < 0.01). Comparisons between the surgery and control groups similarly showed a statistically significant difference (*p* < 0.01). The group examined 5 days after surgery was not significantly different from the group examined 14 days after surgery (*p* = 0.29).

At day 1, the expression of TIMP-2 began to increase until it reached a peak at day 5, and then gradually decreased until day 28, to values still higher than the values measured for the control group. The increase in TIMP-2 levels in conjunctival and subconjunctival tissues was statistically significant (*F* = 145.34, *p* < 0.01). Comparisons between the surgery and control groups similarly showed a statistically significant difference (*p* < 0.01). The group examined 7 days after surgery was not significantly different from the group examined 14 days after surgery (*p* = 0.42).

### 3.6. Localization of MMP-2 and TIMP-2

**Figure 4:**
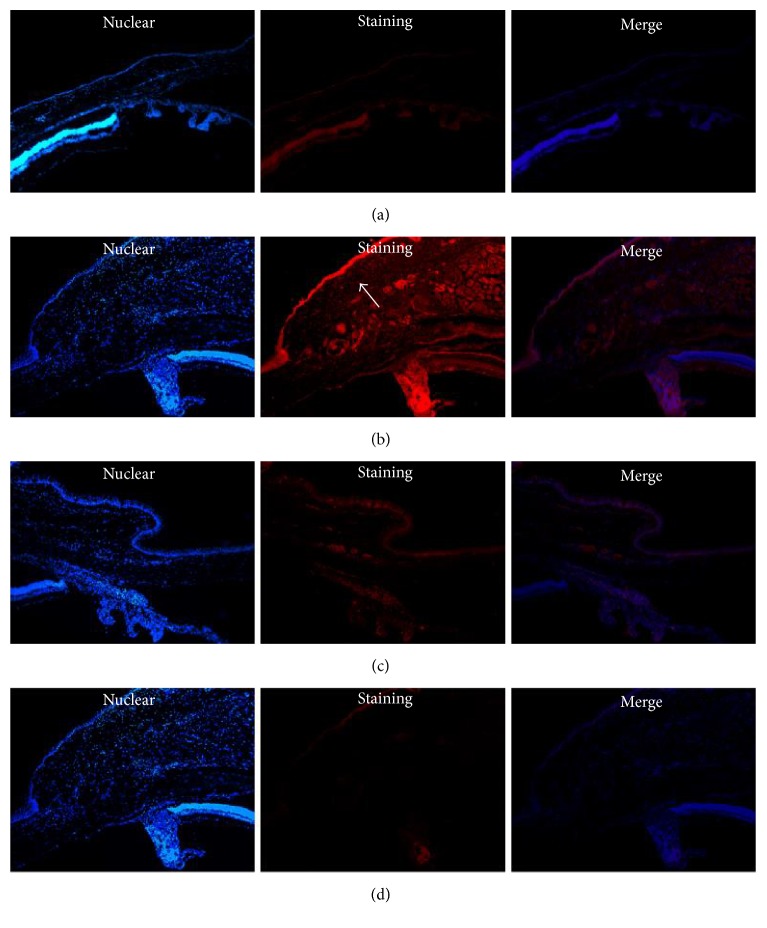
The expression of MMP-2 in the epithelial layers of the normal conjunctiva and in the conjunctival filtration area (immunofluorescence staining, 100x amplification). (a)* C*ontrol group: staining of MMP-2 only in the epithelial layers of the normal conjunctiva was weak. (b, c) At day 7 and at day 28: abundant staining for MMP-2 was detected in both the epithelial and stromal layers of cystic filtering blebs. (d) Negative controls (at day 7: absence of primary antibody) showed negative staining (↗ indicates staining).

**Figure 5:**
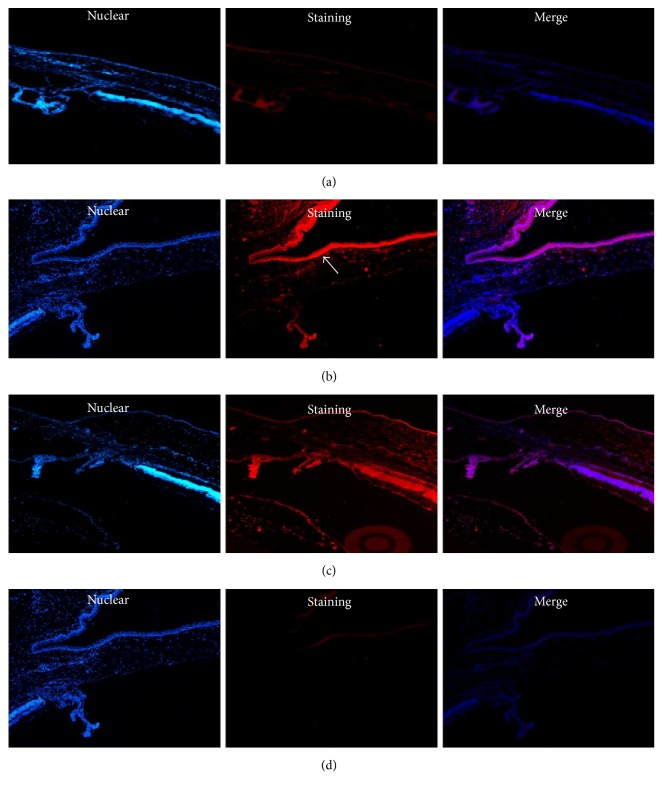
The expression of TIMP-2 in the epithelial layers of the normal conjunctiva and in the conjunctival filtration area (immunofluorescence staining, 100x amplification). (a)* C*ontrol group: staining of TIMP-2 only in the epithelial layers of the normal conjunctiva was weak. (b, c) At day 7 and at day 28: abundant staining for MMP-2 was detected in both the epithelial and stromal layers of cystic filtering blebs. (d) Negative controls (at day 7: absence of primary antibody) showed negative staining (↗ indicates staining).

Similar to our western blot experiments, we observed a pronounced increase in the expression of MMP-2 and TIMP-2 by immunofluorescence staining. The distributions of MMP-2 and TIMP-2 were essentially the same: abundant staining was detected in both the epithelial and stromal layers of the cystic filtering blebs; however, it was detected only in the epithelial layers of the conjunctiva in control group (Figures 4 and 5). After 7 days, the connective tissue of the bulbar conjunctiva was loose, the subconjunctival cells were actively proliferating, and the conjunctival blebs were elevated. At 28 days, the conjunctival blebs almost disappeared.

## 4. Discussion

Rats have previously been used as surgical models for studying conjunctival filtering blebs. (1) Sheridan et al. used a 30-gauge needle to form a fistula through the limbal sclera into the anterior chamber in Lewis rats [17]. Using black C57B1/6 mice, Mietz et al. created a fistula from the subconjunctival space to the anterior chamber by external penetration with a 25-gauge needle through the bulbar conjunctiva [18]. This method can be done easily and repeatedly; however, it cannot completely simulate filtering surgery. (2) Ma et al. cut about 1.0 × 1.5 mm of full-thickness tissue from the limbus, followed by a peripheral iridectomy to set up the filtering bleb [19]. This operation is difficult to conduct in rats, as they tend to bleed excessively during the procedure. (3) Sherwood et al. [15] performed filtering surgeries on Sprague-Dawley rats by inserting a 29-gauge needle into the anterior chamber and introducing a 30-gauge silicone cannula through a penetrating scleral tunnel to drain aqueous humour to the subconjunctival space. By employing insertion of a tube into the anterior chamber and drainage of aqueous humour into the subconjunctival space, this method more closely resembles clinical glaucoma implantation and was the favoured method for characterizing progressive bleb failure. Therefore, we used this method to develop a conjunctival bleb model. The whole process causes a typical wound healing scarring response, similar to the wound healing process following glaucoma surgery in humans.

We observed significant differences in the degrees of elevated conjunctival blebs for all experimental eyes after postoperative day 1. Flat conjunctival blebs were visible by days 7–17, with a mean time of 12 days, which is longer than the mean time described by Sheridan et al. [17] The difference in times to failure may be related to the diameter of the silicone tube implants, because we used a 25-gauge needle, thicker than the 30-gauge needle used in the Sheridan study, resulting in a larger diameter outflow tract. This means that the closure of the incisions in our study is slightly longer, resulting in a longer survival of the filtration bleb. Another explanation for the difference may be the ages of the rats, individual wound healing responses, intraoperative treatments, and other related operations. In this study, all surgeries were performed by a researcher who did not contribute to postoperative observations, reducing the experimenter bias for those evaluating the experimental factors between samples.

In research of filtering surgery in owl monkeys, Desjardins et al. observed that fibroblasts had proliferated along the walls of the opening by day 6 [20]. In the days 7–14 phase, the proliferation of fibroblasts was observed at the surface of the sclera, and there was macrophage migration, angiogenesis, and collagen deposition; after 14 days, the authors observed wound closure. In another study of wound healing, Shima et al. performed glaucoma filtration surgery in rabbits [11]. At 7 days after surgery, a significant number of macrophages were observed where collagen fibrils were deposited, whereas neutrophils had disappeared. After 14 days, fibroblasts became the dominant cell type, and the wound was closed. In our study, histological observation revealed that the wound was replaced by an abundance of fibroblasts, and there was an increase in new collagen at 7–14 days after the operation. Significant deposition of dense collagen fibres can be seen after 28 days, forming the scar. These histological changes during the formation and scarring of conjunctival filtering blebs were consistent with observations from previous research. Taken together, the data demonstrate that the critical phase of wound healing is within two weeks of scar formation after surgery.

The localization of MMP and TIMP expression around blebs has previously been reported. Yang et al. found MMP-2 and MMP-9 expression in the normal conjunctival tissue [21]. McClusky et al. [10] detected MMP-1, MMP-2, MMP-3, and TIMP-2 surrounding the conjunctival tissue of patients who underwent glaucoma drainage implant surgery. Mathalone et al. [22] detected abundant MMP-9 and MMP-2 staining in the epithelial and stromal layers of the cystic leaking blebs, particularly in hypercellular and more vascularized stromal layers. They further found TIMP-1 and TIMP-2 expression in both the epithelial and stromal layers of cystic filtering blebs. In this study, we observed a pronounced increase of MMP-2 and TIMP-2 expression in the epithelial and stromal layers of cystic filtering blebs. We observed that the formation of conjunctival filtering blebs and the scarring process in rats are accompanied by the dynamic expression of MMP-2 and TIMP-2. At postoperative day 1, MMP-2 levels were reduced to about half of their typical levels. It is likely that the oedema around the incision site and the filtration area may have caused a loss of mobile MMP-2. We also speculate that the filtering surgery might have disrupted the normal ECM, leading to activation of MMP-2 to degrade the abnormal ECM. At postoperative day 3, the exacerbation of inflammation in the filtration area increased MMP-2 expression, which might be explained by the release of a variety of cytokines and growth factors. At postoperative day 28, filtering tissue is characterized by scattered distribution of fibroblasts and dense collagen scars. The entire repair process following traumatic inflammation takes 6–8 weeks, which explains why the expression of MMP-2 remains slightly elevated relative to the baseline level.

Wound scarring after filtration surgery occurs by a series of complex and dynamic biological reactions [23]. MMP-2 was more strongly expressed in the surgical wound at 3–7 days after surgery in this experiment, with expression peaking at 7 days. This represents the early phase of wound healing, when infiltration by phagocytes (e.g., neutrophils, macrophages) occurs concurrent to removal of blood clots, plasma fibronectin, and cellular or matrix debris, while the production of a new ECM by fibroblasts begins. We observed that the most active fibroblast proliferation and migration were at 5–7 days after surgery. At this stage, MMP-2 is secreted by macrophages and fibroblasts, increasing the tissue content of MMP-2, and the proliferation of fibroblasts leads to peak expression levels of MMP-2. Similar to MMP-2, we observed an increase in TIMP-2 expression during this early phase, at a time when the blebs were elevated.

At 7–14 days after surgery, the wound was replaced with an abundance of fibroblasts and increased deposits of newly synthesized collagen. The fibroblast population was then reduced, replaced with lens fibre cells, whereas MMP-2 and TIMP-2 levels continued to decrease. At this time, the majority of the filtering blebs disappeared, with the function bleb survival rate falling from 77.7% at day 7 to 20% at day 14. Mature and dense collagen appeared after 28 days. The expression of MMP-2 was higher than TIMP-2, leading to degradation of the collagen type IV and lamin ECM. The remodeling of the ECM resulted in the eventual formation of a fibrous scar. At this time, blebs had almost completely disappeared.

Filtering surgery can damage the structures of the conjunctiva and subconjunctival tissue, which stimulates fibroblasts, as mediated by macrophages, neutrophils, and inflammatory cytokines [24, 25]. These molecules are involved in complex processes, leading to further ECM secretion and remodeling. Any overwhelming activity within this process can cause overstimulation of fibroblasts, formation, and accumulation of ECM. MMPs participate in the process of remodeling ECM and the contraction of matrix, which eventually resulted in scarring [26]. Mitomycin C (MMC) has enhanced the surgical success rate of trabeculectomy through inhibition of Tenon capsule fibroblast proliferation. However, use of MMC was associated with conjunctival epithelial damage, and it is characterized by nonhealing leaking blebs and the risk of an avascular bleb and bleb infection. During the many trials to find a more physiologic, alternative agent, previous studies have suggested that MMPs may play an important role [12–14], where the healing response after surgery could be modulated by inhibiting the effects of MMPs. Activated fibroblasts and macrophages can secrete MMPs during tissue repair, with the increased expression of TIMP-1 and TIMP-2, the endogenous inhibitors of MMPs, further suppressing the activity of MMPs [27, 28]. In this study, we found that the balance between the expressions of MMP-2 and TIMP-2 is closely related to the wound healing response following filtering surgery, which may be one of the key factors interfering with the fibrosis of bleb. However, a MMP inhibitor is a nonspecific target agent and we did not know the exact mechanism of modulating the wound healing process in filtering blebs. In the following study, we will try to increase the expression of TIMP-2 by gene transfection in or after operation, resulting in specific inhibition of MMP-2 expression, and enhance the success rate of filtering surgeries.

## 5. Conclusion

In this study, we were able to observe the most active wound healing response following filtering surgery. From the histological observations of conjunctival filtering blebs, we identified that the critical time for scar formation was within two weeks after surgery. In this period, conjunctival filtering blebs changed gradually from being elevated to flat, until they disappeared. The dynamic changes of MMP-2 and TIMP-2 levels surrounding the conjunctival filtering blebs could be observed during the entire process. Our results suggest that regulatory intervention should be adopted within 14 days, and this is the key to creating a successful filtration bleb, which provides the basis for prevention of future scarring. We further suggest the possibility of inhibiting scar formation by interfering with the expression of MMP-2.

## Figures and Tables

**Table 1:** Intraocular pressure changes after filtration surgery in rats.

Groups	Cases	IOP (mmHg)
Control group	6	7.091 ± 1.397
At day 1	6	4.152 ± 0.656
At day 3	6	4.529 ± 1.077
At day 5	6	5.063 ± 0.904
At day 7	6	6.975 ± 1.343
At day 14	6	7.089 ± 1.054
At day 28	6	7.103 ± 0.882

**Table 2:** Dynamic observation of survival time about filtration surgery in rats.

Postoperative days	Cases	Functional blebs	Survival rates (%)
1	54	54	100
3	45	45	100
5	36	36	100
7	27	21	77.7
14	18	4	22.2
28	9	0	0

**Table 3:** The expression of MMP-2 in conjunctival and subconjunctival tissue of filtration area by western blot (average gray value, $\overset{-}{x}\pm S$, *n* = 6).

Group	Cases	Average gray value
Control group	6	121.67 ± 4.37
At day 1	6	63.17 ± 6.11
At day 3	6	183.67 ± 5.61
At day 5	6	196.83 ± 9.72
At day 7	6	230.50 ± 8.48
At day 14	6	192.67 ± 6.12
At day 28	6	172.33 ± 8.43

**Table 4:** The expression of TIMP-2 in conjunctival and subconjunctival tissue of filtration area by western blot (average gray value, $\overset{-}{x}\pm S$, *n* = 6).

Group	Cases	Average gray value
Control group	6	102.50 ± 6.25
At day 1	6	125.00 ± 6.90
At day 3	6	162.67 ± 7.00
At day 5	6	232.00 ± 11.03
At day 7	6	189.00 ± 8.44
At day 14	6	194.67 ± 8.64
At day 28	6	150.50 ± 6.41
